# Obesity and Women's health across the lifespan: A clinical review of life Stage–Specific challenges and treatment considerations

**DOI:** 10.1016/j.obpill.2026.100321

**Published:** 2026-08-14

**Authors:** Melanie G. Cree, Rebecca Dunsmoor-Su, Samar R. El Khoudary, Maria Hurtado Andrade, Ekta Kapoor, Genevieve Neal-Perry, Nanette Santoro

**Affiliations:** a Department of Pediatrics, University of Colorado School of Medicine, 13199 East Montview Boulevard, Aurora, CO, 80045, USA; b Seattle Clinical Research Center, 633 Yesler Way, Seattle, WA, 98104, USA; c Gennev, 11335 NE 122nd Way, Kirkland, WA, 98034, USA; d Department of Epidemiology, Virginia Commonwealth University School of Public Health, 830 E Main Street, Richmond, VA, 23219, USA; e Departments of Comprehensive Center and Endocrinology, Mayo Clinic, 4500 San Pablo Rd S, Jacksonville, FL, 32224, USA; f Department of Internal Medicine, Mayo Clinic, 200 1st Street SW, Rochester, MN, 55905, USA; g Department of Obstetrics and Gynecology, University of North Carolina School of Medicine, 321 S Columbia St, Chapel Hill, NC, 27599, USA; h Department of Obstetrics & Gynecology, University of Colorado School of Medicine, Research Complex 2, 12700 E 19th Avenue, Aurora, CO, 80045, USA

**Keywords:** Menopause, Obesity, Obesity medications, Obesity-related comorbidities, Reproductive age, Women's health

## Abstract

**Background:**

Obesity prevalence among females in the United States continues to rise across the lifespan, imposing a substantial burden on individuals and the health care system through an increasing number of preventable, excess weight-attributable disabling diseases and mortality. A clearer understanding of the impact of obesity and treatment interventions on outcomes for reproduction, cardiometabolic risk, musculoskeletal health, and mental health at various life stages is critical to optimizing management strategies for women with obesity.

**Methods:**

This narrative clinical review synthesizes published literature and expert perspectives regarding obesity-related risk factors, health outcomes, and treatment considerations related to obesity in women, including the impact of obesity medications across the lifespan.

**Results:**

Adiposity influences reproductive, cardiometabolic, musculoskeletal, and mental health outcomes across the female lifespan. During adolescence, early pubertal onset and metabolic dysfunction are common. In reproductive years, infertility, polyendocrine metabolic ovarian syndrome, and pregnancy complications are prominent. Perimenopause is associated with vasomotor symptoms, sleep disruption, and increased cardiovascular risk. In older age, risks for cancer, functional decline, and frailty increase. Pharmacologic therapies, including incretin-based agents, demonstrate efficacy in weight reduction and cardiometabolic improvement, although life stage-specific safety and outcome data remain limited.

**Conclusion:**

Obesity management in women requires individualized, life stage-specific approaches integrating lifestyle interventions and pharmacotherapy. Additional research is needed to clarify long-term outcomes and optimize treatment strategies across the lifespan.

## Graphical abstract

Recommendations for treating women with obesity based on life stage. CVD, cardiovascular disease; HCP, health care practitioner; PMOS, polyendocrine metabolic ovarian syndrome.

**Figure:**
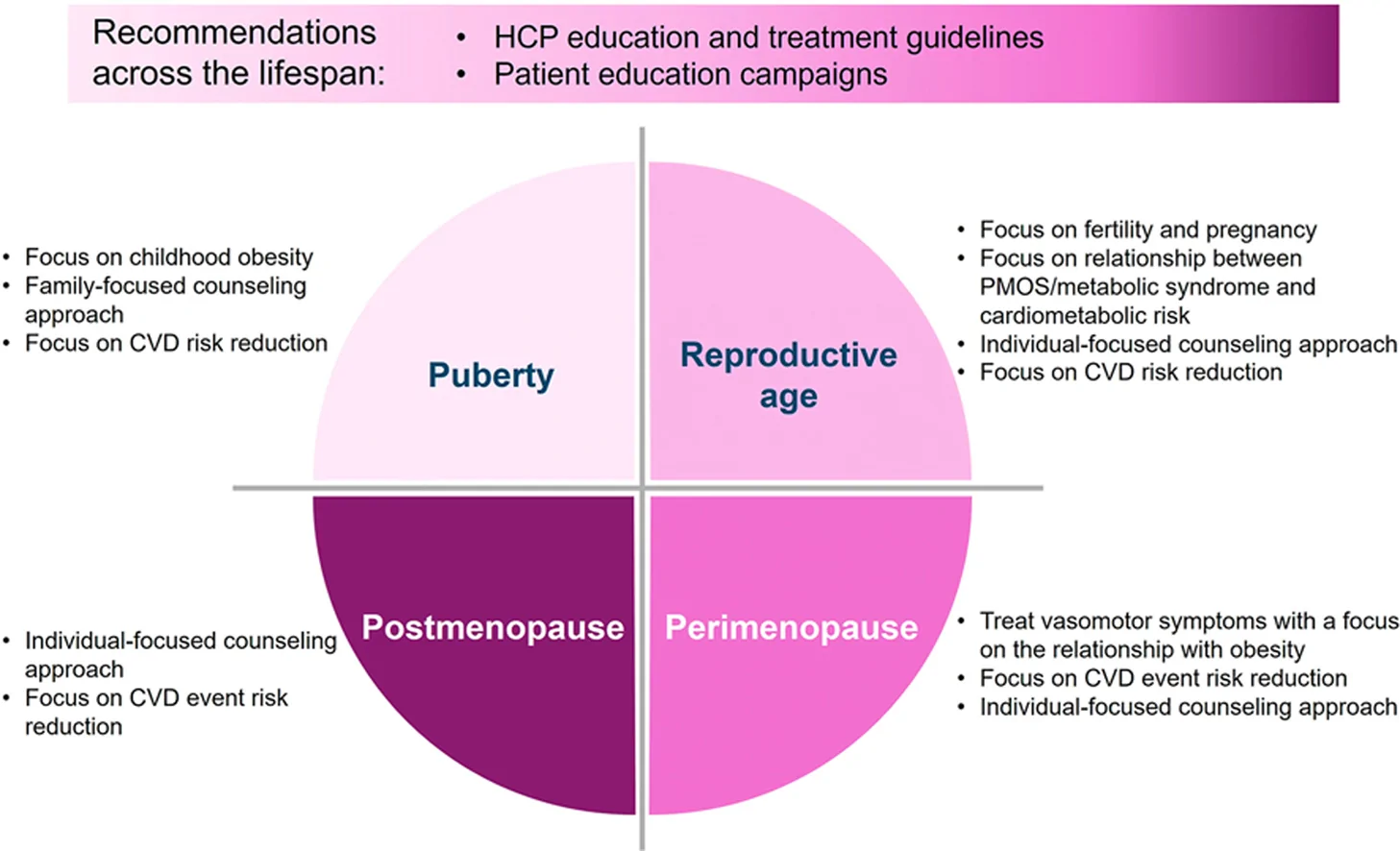

## 1 Introduction

Obesity is one of the most common chronic diseases in the United States. An estimated 41.8% of women aged ≥25 years had obesity in 2021, and this percentage is projected to increase to 58.8% by 2050 [1]. Globally from 1990 to 2021, the absolute number of deaths and disability-adjusted life-years (DALYs) attributable to high body mass index (BMI) increased significantly among women, while age-standardized rates remained stable [2]. These trends indicate a rising global burden of obesity-related DALYs, driven by the increasing prevalence of overweight and obesity in women rather than increased relative risk and highlight the need for sex-specific intervention strategies [2]. Obesity can contribute to adverse health conditions, and the propensity for weight gain and obesity-related comorbidities (ORCs) can influence female-specific physiologic changes across the lifespan [2,3].

A clearer understanding of the changing risk factors and associated health outcomes for obesity throughout the female lifespan is critical to improving treatment strategies. While ORCs are well documented in the general population, the differing presentations that occur throughout the female reproductive life stages require further examination, and knowledge gaps related to obesity medications (OMs) for the management of ORCs remain. For example, the effects of OMs on long-term changes in metabolic health in adolescents are unknown. Additionally, the safety of OMs during fertility treatments or pregnancy is not well understood [4]. OM use in perimenopausal and menopausal women has increased; however, the impact of rapid weight reduction on the perimenopause and menopause experience, muscle mass, mental health, urinary incontinence, and bone health is not fully elucidated and requires further investigation [5]. Lastly, knowledge gaps remain regarding the impact of OMs on physical function/frailty, cancer, and dementia among postmenopausal and older individuals.

This review examines how obesity differentially affects women at each life stage by identifying life stage–specific risk factors and health outcomes associated with obesity and summarizes the impact of OMs on subclinical and clinical outcomes across these life stages. Finally, this review aims to disseminate provider insights regarding the holistic management of excess weight in women, which were shared during the Women's Health and Weight Management virtual, asynchronous advisory board sponsored by Novo Nordisk Inc and conducted during May to June 2024.

## 2 Methods

This narrative clinical review summarizes published literature and expert perspectives regarding obesity-related risk factors, obesity-related comorbidities, and treatment considerations in women across the lifespan. Relevant literature was identified from peer-reviewed publications from the last ten years (2015–2025) and supplemented by insights from a women's health advisory board. Only papers published or translated into English were reviewed. The final reference list was generated on the basis of relevance to the broad scope of this clinical review.

## 3 Risk factors across the female lifespan

Women experience hormonal and physical changes throughout the lifespan, and risk factors for obesity or overweight may co-vary across life stages. In adolescence, early-onset puberty is associated with relatively higher BMI in girls [3]. During sexual maturation and reproductive age, pregnancy typically results in weight gain and is accompanied by increased risk of postpartum overweight or obesity [3]. The menopause transition and older age are marked by estrogen decline and present distinct risk factors for weight gain, including changing body composition, decreased energy expenditure, and lifestyle changes [3,6].

## 4 Obesity-related comorbidities (ORCs) that affect women in multiple life stages

Some ORCs affect women through multiple life stages. For example, in premenopausal women, obesity is associated with increased risk of colorectal and ovarian cancers [7,8]. Additionally, women with obesity who experience abnormal uterine bleeding and polyendocrine metabolic ovarian syndrome (PMOS) have a higher risk of uterine cancer [7,9]. Obesity is also associated with breast, uterine, and ovarian cancers in postmenopausal women [7]. In women with obesity, insulin resistance, hypertension, unopposed estrogen exposure, and anovulation increase the risk of endometrial cancer [10]. Obesity is also a risk factor for pelvic floor disorders, including lower urinary tract symptoms, stress urinary incontinence, overactive bladder, and pelvic organ prolapse [11]. Although there is no consensus, obesity and overweight may negatively influence sexual function. Deniz and colleagues found that sexual function, as measured by the Female Sexual Function Index, is decreased in women with obesity and infertility, and this dysfunction is positively correlated with BMI [12]. Obesity and overweight are also related to suboptimal mental health throughout the lifespan. Biopsychosocial factors, including body image, eating disorders, weight stigma, substance abuse, and neuroendocrine alterations, are implicated in depression associated with obesity [7].

## 5 Age-specific concerns

### 5.1 Sexual maturation and puberty

**Fig. 1:**
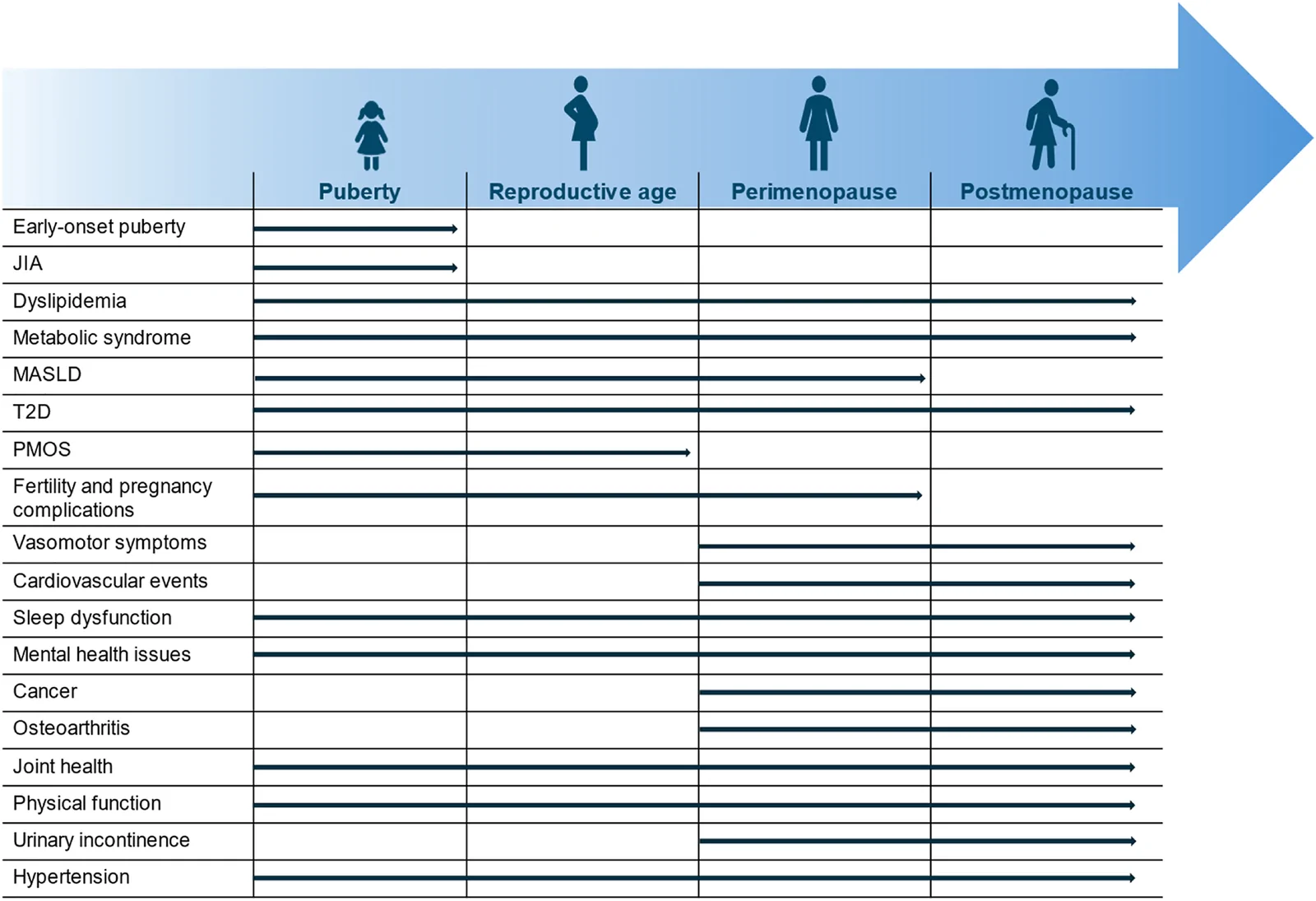
Age-specific ORCs in women. JIA, juvenile idiopathic arthritis; MASLD, metabolic dysfunction–associated steatotic liver disease; ORCs, obesity-related comorbidities; PMOS, polyendocrine metabolic ovarian syndrome; T2D, type 2 diabetes.

Certain ORCs may be more prevalent at specific life stages in females (Fig. 1). In childhood, persistent overweight or obesity may trigger earlier puberty onset and prolong the transition, attributed to factors such as hypothalamic inflammation from short-term overnutrition or morphologic and physiologic microglia cell alterations, adrenal androgen secretion, elevated leptin and insulin levels, and increased concentrations of adipose tissue-produced estrone [13]. Precocious puberty in female children is associated with hyperlipidemia characterized by elevated triglycerides and total and low-density lipoprotein cholesterol compared with healthy controls as well as increased risk for sexual abuse [14,15].

Obesity in adolescence is a strong predictor of early and more severe cardiometabolic disease, including early mortality [16]. During puberty, there is an increase in insulin, thought to arise from increased secretion of growth and sex hormones. This excess insulin, when combined with insulin excess from obesity, contributes to ORCs, including metabolic syndrome, dyslipidemia, hypertension, metabolic dysfunction–associated steatotic liver disease (MASLD), type 2 diabetes (T2D), and a more severe presentation of PMOS. When PMOS is present, the risk of these ORCs is further increased [16]. The development of MASLD with fibrosis in children or adolescents is thought to contribute to the increased risk of requiring hepatic transplantation [7]. Adolescent T2D is more common in females and more aggressive than in adults [17]. It is also associated with a wide range of negative outcomes, including increased severity of obesity, greater insulin resistance, more frequent dyslipidemia, worse pregnancy outcomes, and earlier and more severe consequences of T2D (eg, retinopathy, neuropathy, and nephropathy) [17]. Other ORCs in this age group include obstructive sleep apnea (OSA), skin changes such as acanthosis nigricans, acrochordons, and greater mood dysregulation [16,18,19]. Obesity is also associated with increased involvement of lower limbs in juvenile idiopathic arthritis (JIA) and may negatively impact both disease course and treatment response. Treatment of JIA with glucocorticoids is commonly associated with hyperglycemia and insulin resistance, which may further contribute to ORCs [20].

### 5.2 Reproductive age

During the reproductive years, common ORCs include PMOS, menstrual cycle irregularities (oligomenorrhea and menorrhagia), and fertility and pregnancy complications (eg, early pregnancy loss, gestational diabetes, hypertension, and preterm birth). In adulthood, women with PMOS may have hyperinsulinemia, which is more severe when accompanied by obesity. The combination of excess testosterone and insulin increases risk for T2D, OSA, elevated blood pressure and serum lipids, and MASLD [21]. The risk of MASLD also increases with increased BMI and is associated with higher incidence of gestational diabetes and early pregnancy loss [7]. Furthermore, MASLD and OSA have potential to negatively affect fetus birth weight and increase risk of preterm delivery [22,23].

Compared with women without obesity, women with obesity and without T2D experience higher rates of infertility due in part to PMOS-related anovulation, poorer oocyte quality, implantation failure related to suspected abnormal endometrium, and poorer pregnancy outcomes [4,[24], [25], [26]]. Compared with women with a BMI of 18.5 to <25, pregnancy complications in women with obesity include higher risk of miscarriage, stillbirth, preeclampsia, gestational diabetes, and venous thromboembolism [27]. Obesity is also associated with increased risk of requiring cesarean delivery and subsequent wound infections and postpartum hemorrhage [27,28]. Pregnancies in women with obesity carry increased risks of fetal growth abnormalities (microsomia and growth restriction) and congenital birth defects [3,4,[24], [25], [26], [27]]. Maternal obesity may also program obesity and other metabolic morbidities in their offspring (Barker hypothesis) [29]. Breastfeeding has been shown to have metabolic benefits for the mother and infant; however, breastfeeding challenges, including lower rates and greater difficulty have been reported in women with obesity [7].

### 5.3 Perimenopause

During perimenopause, commonly experienced ORCs include cardiometabolic disease, vasomotor symptoms (VMS), sleep impairment, and mental health issues. During the menopause transition, risk of cardiovascular disease (CVD) events also accelerates and is linked to changes in sex hormones, alterations in body composition characterized by increased visceral adiposity, and adverse changes in vascular health and lipids and lipoproteins [30]. VMS worsening during perimenopause is hypothesized to result from disrupted thermoregulation and heat dissipation due to excess adiposity [31]. VMS associated with perimenopause contribute to sleep disruption—an added risk factor for obesity [32]. In the general population, there is a bidirectional link between overweight/obesity and emotional distress, which may be influenced by adipokines contributing to systemic inflammation and affecting mood regulation. Mood disorders related to perimenopause may also lead to increased generation of cytokines and free radicals and contribute to fat accumulation [33].

### 5.4 Postmenopause and older age

During the postmenopausal stage, common ORCs include cancer, osteoarthritis, physical dysfunction, and urinary incontinence. Obesity is associated with an increased incidence of endometrial, renal, esophageal, ovarian, and breast cancers in women during this life stage [7]. In postmenopausal women with obesity, there is also an association between estrogen and progesterone receptor-positive breast cancer [34]. Increased load on weight-bearing joints and local and systemic inflammatory mediators that are associated with excess adiposity may play significant roles in the development of osteoarthritis in this population [35]. Higher perivascular adipose tissue in midlife is associated with slower gait speed in later life and may contribute to decreased physical function [36]. Women with obesity in menopause or older age are more likely to experience urinary incontinence due in part to increased abdominal and/or bladder pressure [33]. Postmenopausal women also experience an increased incidence of OSA [32]. While the prevalence of MASLD is typically higher for men than women during reproductive years, this difference plateaus after 50 years of age, as women are 2.4 times more likely to develop MASLD after menopause, likely related, in part, to the decline in estrogen levels [37]. In conjunction with other comorbidities—such as dyslipidemia, visceral obesity, and T2D—MASLD also contributes to an increased cardiovascular risk in postmenopause [37]. The effects of hypoestrogenism on lipid metabolism, energy homeostasis, insulin resistance, and body fat distribution, and the effects of inflammation in blood vessels, among other mechanisms, are likely contributors to this increased risk [6].

## 6 Intervention strategies

Intervention strategies for obesity largely fall into the treatment domains of nutrition, physical activity, behavior modification, pharmacotherapy, or endoscopic/surgical procedures [3,[38], [39], [40]]. Nutrition interventions include adjusting macronutrient composition, dietary patterns, and meal timing as well as increasing micronutrient density [3]. While physical activity alone is rarely effective for sustained weight reduction, aerobic and resistance exercise can help maintain total energy expenditure, attenuate loss of muscle mass, and improve physical function during weight reduction [3]. Pharmacotherapy interventions approved for long-term weight management include non-incretin medications (ie, phentermine/topiramate and naltrexone/bupropion) and incretin-based therapies such as glucagon-like peptide-1 receptor agonists (GLP-1 RAs) and dual glucose-dependent insulinotropic polypeptide (GIP)/GLP-1 RAs [3]. Sympathomimetic monotherapies approved for short-term use (up to 12 weeks) include phentermine, diethylpropion, benzphetamine, and phendimetrazine [3,41]. Several medications shown to reduce weight may also be used off-label for obesity treatment, including metformin, sodium-glucose cotransporter-2 inhibitors (SGLT2i), and amylin antagonists [3]. Although phentermine and topiramate have more than 30 years of safety data, non-incretin OMs produce less significant weight reduction compared with the most effective incretin-based medications [3]. GLP-1 RAs induce long-term protective effects on cardiovascular health, renal outcomes, and adverse events in individuals with obesity and without T2D [42,43]. They are also approved to treat CVD, MASLD, and OSA [42,43]. For two decades, GLP-1 RAs have demonstrated safety and tolerability in treating T2D, with low risk of hypoglycemia in patients with and without T2D [44,45].

Bariatric surgeries induce weight reduction through various restrictive and metabolic effects; however, caution is required to avoid rapid weight reduction, which can cause hypogonadotropic hypogonadism [3,46]. OMs are not approved for use during pregnancy or breastfeeding; waiting 12–24 months after bariatric surgery before conceiving is recommended to avoid adverse effects of rapid maternal weight reduction on fetal development. These factors should be considered when treating women with obesity [3]. Recommendations on timing of discontinuation of OMs prior to conception are evolving to best balance safety and maternal and fetal well-being. However, given the long half-lives of these medications, for them to be completely cleared before attempting conception requires discontinuation up to 4–8 weeks, depending on the medication [4].

## 7 Impact of OMs on obesity management and subclinical and clinical outcomes by life stage

OMs have demonstrated effectiveness in reducing overweight and obesity across life stages in women. For example, a post hoc analysis of the randomized, placebo-controlled SURMOUNT-1, -3, and -4 trials examined the effect of tirzepatide, a combined GIP/GLP-1 RA, on weight, waist circumference, and waist to height ratio of women with obesity or overweight across premenopause, perimenopause, and postmenopause stages. Regardless of reproductive stage, treatment with tirzepatide was associated with significant reductions in all measures [47]. Data from a post hoc analysis of the SELECT trials, stratified by sex, showed that the GLP-1 RA semaglutide is associated with greater reductions in weight and waist circumference in women compared with men [48].

Metformin, an insulin sensitizer, reduces caloric intake by decreasing insulin concentrations and causing mild nausea, thereby reducing hunger [49]. When combined with a carbohydrate-modified diet, metformin enhances 12-month weight reduction and improves body composition in an ethnically diverse normoglycemic, hyperinsulinemic population of women with midlife weight gain [50]. Phentermine promotes weight reduction (5%–10%) by reducing food intake through increased satiety but is contraindicated in patients with CVD or high CVD risk [3]. For patients with obesity and comorbidities that are treatable with bupropion or naltrexone, bupropion-naltrexone may promote weight reduction of 4%–5%; however, its cardiovascular safety profile is not well established, and it can lower the seizure threshold [3].

### 7.1 Sexual maturation and puberty

OMs decrease ORCs that are prominent during puberty. The American Academy of Pediatrics recommends OMs for the treatment of obesity in adolescents [51]. Multiple OMs are approved for use in adolescents, including phentermine, combined phentermine/timed-release topiramate, and the GLP-1 RAs liraglutide and semaglutide [51]. More adolescents experienced ≥5% weight reduction with oral combined phentermine/extended-release topiramate compared with injectable liraglutide, and the amount of weight lost was greater [51]. Semaglutide induced the greatest weight reduction, although 24% did not lose at least 5% of weight. Liraglutide is effective for weight reduction in children aged 5–11 years [51]. Given the relationship between obesity and early onset of puberty, use of OMs could alter the timing of puberty in children with obesity [13].

OMs may decrease insulin resistance and related cardiometabolic disease, although the improvement is lower in adolescents than in adults, likely due to increased growth hormone secretion during puberty [17]. In adolescents, semaglutide results in greater reductions in cardiometabolic risk factors, including hemoglobin A1c levels, waist circumference, lipids (excluding high-density lipoprotein cholesterol), and alanine aminotransferase compared with placebo [52]. The degree of weight reduction induced by OMs, including phentermine plus topiramate, naltrexone plus bupropion, liraglutide, and semaglutide, generally correlates with the effect size on lowering lipid levels [53]. Incretin-based therapies are approved in adults for select ORCs, including MASLD, OSA, and CVD. Because these conditions are present in adolescents, further condition-specific treatments may also become available for this population.

### 7.2 Reproductive age

Risk for hypertension can increase in reproductive-aged women, particularly during pregnancy. While metformin may be prescribed in pregnant individuals and is sometimes used off-label for obesity treatment, OMs are not approved for use during this time [3]. However, reducing risk factors before pregnancy can improve outcomes. For example, treatment with tirzepatide or semaglutide reduces both systolic and diastolic blood pressure in non-pregnant people [54,55].

Behavioral weight reduction interventions have not shown benefit for improving fertility or live birth rates in women with obesity without PMOS, possibly because the weight reduction is not sufficient with behavioral methods to improve outcomes [4,56,57]. Additionally, the stress of behavioral weight reduction may contribute to reproductive dysfunction [4]. While available data show mixed results, OMs may improve symptoms related to PMOS and menstruation and lead to better fertility outcomes. In women with PMOS, treatment with the GLP-1 RA exenatide decreases ovarian dysfunction and free testosterone levels while improving ovulation rates [58]. In a study of women with PMOS, BMI >30 and < 45, and no diabetes, the following therapies reduced free androgen index: the SGLT2i dapagliflozin; dapagliflozin coadministered with once-weekly exenatide; dapagliflozin with extended-release metformin; and phentermine/extended-release topiramate [59]. Exenatide-mediated improvement in insulin sensitivity suggests that GLP-1 RAs may exert these effects independent of weight reduction [58]. Some studies of the GLP-1 analogue liraglutide have shown decreases in testosterone and improved rates of ovulation [60]. Six-month treatment with semaglutide increases ovulation in women with obesity and PMOS, and combination therapy with metformin lowers testosterone more than metformin alone [61,62].

In women with obesity and PMOS resistant to first-line reproductive treatment and lifestyle modification, 12 weeks of preconception treatment with low-dose liraglutide combined with metformin increases in vitro fertilization pregnancy rates and spontaneous pregnancies more than metformin alone [63]. A secondary analysis of two randomized trials in women with obesity and PMOS found a significant improvement in ovulation rate and live birth rate with delayed infertility treatment following lifestyle modification, including weight reduction, compared with immediate infertility treatment [56]. The impact of OMs on live births is still unclear, as interventions in most large randomized controlled trials have included diet and exercise as well as orlistat, which is not commonly prescribed because of its adverse gastrointestinal side effects and lower efficacy in reducing weight compared with other OMs [4,41].

A phase 3 trial of semaglutide and preliminary trials of the OMs tirzepatide and survodutide (a dual glucagon and GLP-1 RA) have shown reductions in metabolic dysfunction–associated steatohepatitis (MASH) [43,64,65]. Based on the phase 3 trial results, semaglutide 2.4 mg was recently approved for treatment of MASH with fibrosis in adults ≥18 years, becoming the first and only GLP-1 RA approved for this indication [66].

### 7.3 Perimenopause, postmenopause, and older age

Some data suggest that OMs may improve clinical outcomes associated with OSA. Weight reduction interventions are associated with reduction in tongue fat, which is a risk factor for OSA. Pharmacological intervention with OMs may facilitate the downstream effects of weight reduction on parameters that influence OSA, such as decreases in blood pressure and low-grade systemic inflammation, that are not fully treated by mechanical treatments such as continuous positive airway pressure (CPAP) [67,68]. For example, tirzepatide significantly improves OSA-related symptoms, including apnea-hypopnea index and hypoxic burden, regardless of concomitant CPAP use [68].

Importantly, given the increased risk of CVD events during the menopause transition, some OMs may also improve cardiovascular outcomes. In the SELECT trial, which included patients with a BMI ≥27 and preexisting CVD without T2D, treatment with semaglutide 2.4 mg was associated with reduced incidence of a composite of death from cardiovascular causes, nonfatal myocardial infarction, or nonfatal stroke [69]. However, OMs that may elevate blood pressure, such as phentermine and diethylpropion, are not recommended for patients with a history of CVD; if prescribed in patients with a history of hypertension, increased monitoring is advised [41].

The impact of treatment with OMs, particularly GLP-1 RAs, on osteoporosis requires further research. Studies in people with obesity have largely focused on the lower doses of GLP-1 RAs used to treat T2D, with inadequate bone parameters as primary end points and short follow-up periods.

## 8 Discussion

To gain an understanding of the holistic management of excess weight in women, the Women's Health and Weight Management virtual, asynchronous advisory board was held from May 31, 2024, to June 14, 2024. Participating advisors comprised physicians with specialties in obstetrics and gynecology and obesity medicine, with a focus on women's health, endocrinology, and infertility and included three authors of this review—Drs. Dunsmoor-Su, Neal-Perry, and Hurtado. In line with the scope of this review, the key objectives of this advisory board were to:

• Understand the journey of women with overweight or obesity and provider approaches to their care
• Gain insights into the treatment goals and challenges associated with managing overweight and obesity in women
• Understand the role of pharmacotherapy in the management of overweight and obesity in women
• Identify the evidence gaps in clinical trials involving women's health

One outcome of the advisory board was conceptualization of this review to disseminate insights and gaps in the current treatment landscape for women with obesity. Key insights from the advisory board are discussed below.

### 8.1 Implications independent of life stage

As reviewed above, some ORCs, including some obesity-related cancers, pelvic floor disorders, sexual dysfunction, and mental health issues, affect women through multiple life stages [7,[10], [11], [12]]. Advisory board participants noted some of the most common ORCs that they encounter are ovulatory dysfunction, diabetes/prediabetes, and mental health issues such as anxiety and depression. Both anxiety and depression are often exacerbated by weight gain. Biopsychosocial factors that influence depression associated with obesity may also fluctuate through life stages [7]. Some of these factors described by advisors include obesity increasing the risk of stigma and bias, employment discrimination, and reduced social equity. Obesity also impacts physical functioning throughout the lifespan, and women with physical and intellectual disabilities may experience increased rates of severe obesity.

### 8.2 Implications that vary by life stage

Risks for other ORCs are influenced by factors that vary across the lifespan and may be more prevalent during specific life stages. Advisors noted that fertility, PMOS, and pregnancy complications, as well as cardiovascular event risk, represent urgent unmet needs in women of reproductive age and in perimenopause, respectively. While preconception weight reduction is associated with improved fertility in women with PMOS, the impact of OMs on live birth rates and perinatal complications or in women with obesity without PMOS is still not well understood [4,41]. There is some evidence for improving PMOS symptoms and menstrual irregularities in women with obesity using GLP-1 RAs, but further research is needed [60]. The advisors noted that menopause and midlife changes are significant factors in weight gain and lead to patient frustration. Furthermore, it was noted that menopause and VMS are associated with greater risk of CVD, and excess adiposity further increases risk. The impact of weight reduction or intervention with OMs on cancer risk, physical function, and urinary incontinence remain understudied in older women and those in menopause.

These unmet needs and knowledge gaps have significant implications for women with obesity and overweight. Advisors noted that managing insulin resistance and obesity presents a significant challenge in patients with PMOS. Additionally, higher BMI is associated with lower pregnancy success rates and higher rates of pregnancy loss. Finally, advisors discussed how OSA and MASLD are underdiagnosed in women despite high prevalence in patients with obesity.

### 8.3 Additional considerations for women with obesity

A more personalized approach and increased physician education are needed for treatment options of all domains (nutrition, physical activity, behavior modification, medication, endoscopic/surgical procedures) [3,38,40]. The most effective intervention strategies for treatment of obesity are likely to include a multidisciplinary approach that considers addressing all obstacles. Also highlighted during the advisory board was the importance of provider education to decrease stigma and bias and to improve the ability to provide informed patient care with treatment options tailored to individual needs. Advisors noted that there is an education and resources gap in the standard of care, as obesity and overweight are not treated as chronic diseases by most physicians, and newer medications are not well understood. Importantly, the role of social determinants of health in treatment interventions must be considered, including the differentiating effects of socioeconomic, racial, and ethnic backgrounds on ORCs. For example, Black women have a higher risk of CVD compared with women from other racial or ethnic backgrounds [70]. Advisors stated that a lack of insurance and the cost of treatment impact access to interventions and their success. Time constraints and childcare are common barriers to physical activity among women [3,71]. Access to fresh fruits and meats and to outdoor spaces for exercise can also impact treatment success [72,73]. Social and/or cultural interpretations and patterns of weight gain, such as value placed on specific body shapes, may influence weight loss management. For example, weight gain and overweight are associated with wealth, higher socioeconomic status, strength, and fertility in some sub-Saharan African, Arabic, and Mexican cultures [3].

Historical misunderstandings and common myths contribute to attitudes toward obesity and weight reduction (Table 1). For example, it was previously thought that a declining metabolic rate due to menopause was primarily responsible for weight gain in women in middle-aged women; however, recent evidence shows that total energy expenditure and basal metabolic rate remain stable until about 60 years of age [75]. Newer evidence also suggests that loss of ‘metabolic flexibility’ related to menopause may be an estrogen-related phenomenon and that menopause is associated with a small increase in fat mass, a decrease in lean body mass, and an increase in visceral fat [76]. Thus, current literature largely supports the premise that weight gain is complex and may reflect a combination of lifestyle changes and altered adipocyte biology in response to the hormone changes that characterize perimenopause and menopause [6].

**Table 1:** Common myths and misunderstandings regarding weight management.

Myth	Facts
OMs are not an effective weight loss intervention	Managing weight reduction using lifestyle modifications alone is difficult for many individuals. OMs are effective as adjuncts to lifestyle changes for achieving and maintaining weight reduction [44,74]
Reducing dietary calories when breastfeeding may lead to reduced milk production	Gradual weight reduction through exercise and moderate caloric restriction does not significantly impact milk production or infant growth [71]
Declining metabolic rate drives weight gain during menopause	Aging and lifestyle changes are the primary drivers of weight gain in midlife [40]
Weight gain is associated with menopause	Weight gain in midlife and later life in women is more related to aging than menopause itself [6]
Obesity is solely due to lack of willpower and discipline to lose weight	Body weight is regulated by complex biological, genetic, psychological, and environmental factors [3]

OMs, obesity medications.

Common beliefs also contribute to behaviors regarding weight gain and loss, such as postpartum weight retention. A cross-sectional survey of postpartum women found that about half of respondents believed that overeating helps provide adequate nutrition to the child, and most thought calorie reduction also reduces milk production [71]. Another common myth relating to nutrition is that adolescents require more calories to support growth. Patients’ perceptions of a lack of efficacy of or access to OMs may also impact attitudes toward these interventions [44,74].

Obesity management is most effective when treatment is individualized to patients and when culturally sensitive interventions are considered. Weight management interventions should focus on aligning with patients' goals, considering long-term health impacts, and incorporating lifestyle and behavioral changes to achieve overall wellness. As noted by advisors, lifestyle interventions by themselves may not be sufficient for long-term success. Considerations should include the patient's life stage. During childhood and puberty, additional focus on addressing obesity during preventive health visits is needed, and OMs should be considered. For women in menopause, midlife changes significantly impact weight gain trajectories. Large trials are needed to demonstrate OM-mediated improvements in menopausal weight issues, cardiometabolic risk, fertility and PMOS, and cancer. Obesity and overweight should be treated as chronic diseases and education on newer OMs and their use should be prioritized.

### 8.4 Recommendations

Given the need to better individualize treatment for women with obesity, recommendations may vary based on life stage (**Graphical Abstract**). Advisors noted that while recommendations such as patient education programs and provider education of treatment guidelines are relevant to each life stage, timing and content of intervention strategies are important, as goals may shift from weight gain prevention in younger women to management of excess adiposity as women age and experience the menopausal transition.

## 9 Conclusion

Obesity across the female lifespan presents unique clinical challenges that require stage-specific management strategies integrating lifestyle interventions, pharmacotherapy, and multidisciplinary care.

**Key clinical takeaways**

• Obesity in women is influenced by unique biological, environmental, and sociocultural factors that vary across life stages and contribute to distinct adiposity-related complications.
• Effective obesity management requires individualized, life stage-specific treatment strategies, supported by increased provider education to address gaps in care and reduce weight stigma.
• Additional research is needed to better define drivers of obesity risk across the lifespan and to evaluate the impact of weight reduction and obesity medications on outcomes that are specific to women.

## Ethics review

This manuscript is a narrative clinical review and did not involve prospective enrollment of human participants, collection of patient data, or interventions requiring Institutional Review Board approval or informed consent.

## Disclosures

MGC has served as a consultant for Novo Nordisk Inc. RDS has served as a consultant for Novo Nordisk Inc., Eli Lilly, and Bayer Pharmaceuticals. She holds RIU in Unified Women's Health. SREK has served as a consultant for Novo Nordisk Inc. MHA has received research support and meeting attendance support from Phenomix Sciences, the National Institutes of Health, and the Mayo Clinic Center for Women's Health Research. She has served as a consultant for Novo Nordisk Inc. and has received honoraria from The Menopause Society and Villanova University. She has served as a safety monitoring board member for “The Role of Patient Capacity in Chronic Kidney Disease Trajectories” (Principle Investigator: K. Boehmer) and has served as an annual planning committee member and spokesperson for The Obesity Society. EK has served as a consultant for Astellas Pharmaceutical, Novo Nordisk Inc., Estetra SRI, Fresenius Kabi, Exeltis Inc., and Wellfound Inc. GNP has received research support from the National Institutes of Health, the Cohen Foundation, and North Carolina State. She has served as a consultant for Astellas Pharmaceutical, Vertex Pharmaceutical, Novo Nordisk Inc., and Bayer Pharmaceutical. She has served on the Finance Committee for FASEB and the Endocrine Society and has served on the Executive Committee for the Society for Reproductive Investigations. NS has served as a consultant for Astellas Pharmaceutical, Ansh Labs, Novo Nordisk Inc., Bayer Consumer Care AG, and Perrigo/Laboratoire HRA Pharma.

## Author contributions

Melanie G Cree contributed to conceptualization and writing – review and editing. Rebecca Dunsmoor-Su contributed to conceptualization and writing – review and editing. Samar R El Khoudary contributed to conceptualization and writing – review and editing. Maria Hurtado Andrade contributed to conceptualization and writing – review and editing. Ekta Kapoor contributed to conceptualization and writing – review and editing. Genevieve Neal-Perry contributed to conceptualization and writing – review and editing. Nanette Santoro contributed to conceptualization and writing – review and editing.

## Declaration of artificial intelligence (AI) and AI-assisted technologies

The authors did not use artificial intelligence or AI-assisted technologies in the generation, interpretation, or scientific writing of this manuscript.

## Funding

10.13039/501100004191Novo Nordisk Inc. provided financial support for the medical writing and covered the open access fee for this project. The authors did not receive any remuneration for their involvement in developing this paper. Novo Nordisk Inc. played a role in identifying and recruiting the authors. Although representatives from Novo Nordisk Inc. reviewed the article for scientific and reference accuracy, they did not influence its content. 10.13039/501100004191Novo Nordisk Inc. also sponsored a virtual, asynchronous expert advisory board on Women's 10.13039/100018696Health and Weight Management to develop relevant questions and identify knowledge gaps. This information was utilized to justify the project and create a draft outline for the manuscript.

## Declaration of competing interests

The authors declare the following financial interests/personal relationships which may be considered as potential competing interests: All Authors reports financial support, article publishing charges, and writing assistance were provided by Novo Nordisk Inc. Melanie G. Cree reports a relationship with Novo Nordisk Inc. that includes: consulting or advisory. Rebecca Dunsmoor-Su reports a relationship with Novo Nordisk Inc. that includes: consulting or advisory. Rebecca Dunsmoor-Su reports a relationship with Eli Lilly that includes: consulting or advisory. Rebecca Dunsmoor-Su reports a relationship with Bayer Pharmaceuticals that includes: consulting or advisory. Rebecca Dunsmoor-Su reports a relationship with Unified Women's Health that includes: equity or stocks. Samar R El Khoudary reports a relationship with Novo Nordisk Inc. that includes: consulting or advisory. Maria Hurtado Andrade reports a relationship with Phenomix Sciences that includes: non-financial support. Maria Hurtado Andrade reports a relationship with National Institutes of Health that includes: non-financial support. Maria Hurtado Andrade reports a relationship with Mayo Clinic that includes: non-financial support. Maria Hurtado Andrade reports a relationship with Novo Nordisk Inc. that includes: consulting or advisory. Maria Hurtado Andrade reports a relationship with The Menopause Society that includes: consulting or advisory, speaking and lecture fees, and travel reimbursement. Maria Hurtado Andrade reports a relationship with Villanova University that includes: consulting or advisory, speaking and lecture fees, and travel reimbursement. Maria Hurtado Andrade reports a relationship with The Role of Patient Capacity in Chronic Kidney Disease Trajectories that includes: board membership. Maria Hurtado Andrade reports a relationship with The Obesity Society that includes: board membership and speaking and lecture fees. Ekta Kapoor reports a relationship with Astellas Pharmaceutical that includes: consulting or advisory. Ekta Kapoor reports a relationship with Novo Nordisk Inc. that includes: consulting or advisory. Ekta Kapoor reports a relationship with Estetra SRI that includes: consulting or advisory. Ekta Kapoor reports a relationship with Fresenius Kabi that includes: consulting or advisory. Ekta Kapoor reports a relationship with Exeltis Inc. that includes: consulting or advisory. Ekta Kapoor reports a relationship with Wellfound Inc. that includes: consulting or advisory. Genevieve Neal-Perry reports a relationship with National Institutes of Health that includes: non-financial support. Genevieve Neal-Perry reports a relationship with Cohen Foundation that includes: non-financial support. Genevieve Neal-Perry reports a relationship with North Carolina State that includes: non-financial support. Genevieve Neal-Perry reports a relationship with Astellas Pharmaceutical that includes: consulting or advisory. Genevieve Neal-Perry reports a relationship with Vertex Pharmaceutical that includes: consulting or advisory. Genevieve Neal-Perry reports a relationship with Novo Nordisk Inc. that includes: consulting or advisory. Genevieve Neal-Perry reports a relationship with Bayer Pharmaceutical that includes: consulting or advisory. Genevieve Neal-Perry reports a relationship with FASEB that includes: board membership. Genevieve Neal-Perry reports a relationship with Endocrine Society that includes: board membership. Genevieve Neal-Perry reports a relationship with Society for Reproductive Investigations that includes: board membership. Nanette Santoro reports a relationship with Astellas Pharmaceutical that includes: consulting or advisory. Nanette Santoro reports a relationship with Ansh Labs that includes: consulting or advisory. Nanette Santoro reports a relationship with Novo Nordisk Inc. that includes: consulting or advisory. Nanette Santoro reports a relationship with Bayer Consumer Care AG that includes: consulting or advisory. Nanette Santoro reports a relationship with Perrigo Company that includes: consulting or advisory. If there are other authors, they declare that they have no known competing financial interests or personal relationships that could have appeared to influence the work reported in this paper.
